# Supplementary material for: Statistical injury prediction for professional sumo wrestlers: Modeling and perspectives
Source: PLoS One. 2023 Mar 17;18(3):e0283242. doi: 10.1371/journal.pone.0283242 (PMC10022813; doi:10.1371/journal.pone.0283242)
Supplement: Attachment — Submitted filename: Response Letter.pdf [file pone.0283242.s002.pdf]

Response Letter

Title: Statistical injury prediction for professional sumo wrestlers: modeling and perspectives

Dear Dr. Kovtun,

We would like to thank the two anonymous reviewers for their insights and helpful comments to improve the clarity and completeness of the manuscript. We have carefully considered the comments and suggestions and addressed them in detail as outlined in the following letter. We hope that the manuscript would be acceptable for publication with these changes addressed by the reviewers.

Thank you very much for your consideration.
Best regards,
S. Ota and M. Kimura

Response to Journal Requirements:

Comment R-1: *Please ensure that your manuscript meets PLOS ONE's style requirements, including those for file naming.*

Response R-1: We have done our best to ensure that the manuscript meets PLOS ONE's style requirements. Since these revisions are not substantive changes, they are not indicated as tracked changes within the manuscript to maintain readability.

Comment R-2: *PLOS requires an ORCID iD for the corresponding author in Editorial Manager on papers submitted after December 6th, 2016.*

Response R-2: This has been done.

Comment R-3: *Please note that PLOS ONE has specific guidelines on code sharing for submissions in which author-generated code underpins the findings in the manuscript. In these cases, all author-generated code must be made available without restrictions upon publication of the work. Please review our guidelines at https://journals.plos.org/plosone/s/materials-and-software-sharing#loc-sharing-code and ensure that your code is shared in a way that follows best practice and facilitates reproducibility and reuse.*

Response R-3: We have provided code for the data analysis as supporting information, which should clarify the procedure for the interested reader.

Comment R-4: *Please review your reference list to ensure that it is complete and correct. If you have cited papers that have been retracted, please include the rationale for doing so in the manuscript text, or remove these references and replace them with relevant current references. Any changes to the reference list should be mentioned in the rebuttal letter that accompanies your revised manuscript. If you need to cite a retracted article, indicate the article's retracted status in the References list and also include a citation and full reference for the retraction notice.*

Response R-4: We have confirmed no retracted papers are cited in the manuscript. In addition, we have added nine references to respond to reviewer's comments.

Comment R-5: *While revising your submission, please upload your figure files to the Preflight Analysis and Conversion Engine (PACE) digital diagnostic tool, https://pacev2.apexcovantage.com/. PACE helps ensure that figures meet PLOS requirements.*

Response R-5: We have uploaded all figure files (*.tif) that are generated by applying the PACE tool to the original figure files (*.eps).

Response to Reviewer #1's comments:

Comment 1-0: *The study proposes a model for predicting the injury occurrences for sumo wrestlers. The new model is a combination of the existing Poisson and the Binomial model that is based in the Hawkes process. The authors are reporting that the model is superior to the existing one in terms of the model selection criteria.*

Response 1-0:

First of all, thank you very much for reviewing the manuscript and giving us valuable comments. Our answer to your comment is given in the following. Almost all the changes in the manuscript (except for deletions) have been highlighted in yellow.

Major comments:

Comment 1-1: *What are the necessary assumption(s) of the proposed model? Are those same as the Poisson model?*

Response 1-1: The proposed model and Poisson process model have common and uncommon assumptions, respectively. Assumptions 1 and 2 on lines 155-157 are the common assumptions for the proposed and Poisson process models. In addition, the proposed model assumes that the likelihoods of *kyujo* occurrences are dependent on all previous *kyujo* occurrences for each sumo wrestler. However, the Poisson process model assumes that the likelihoods of *kyujo* occurrences are homogeneous and independent of previous *kyujo* occurrences for each sumo wrestler. In the revised manuscript, we have explicitly mentioned those points.

Revised area in the manuscript:

- Lines 146-148, 158, 160-161, 170-172

Comment 1-2: *If a model is more than 2 AIC units lower than another, then it is considered significantly better than that model. In Table 1, the AIC for the proposed model is not so and hence may not be the good choice over the Poisson model.*

Response 1-2: According to Burnham and Anderson [43,44], if the difference between the AIC for a model and the minimum AIC is greater than 10, it indicates that the model having the minimum AIC is considered significantly better. In Table 1, the AIC for the proposed model is the minimum value, and the difference between the AIC for the Poisson process model and the minimum AIC is $-1058.03 - (-1267.93) > 10$. Hence, we have mentioned that the proposed model is a good choice over the Poisson process model.

Revised area in the manuscript:

- Lines 251-255

- References [43, 44]

Comment 1-3: *Generally, a large confidence interval indicates the sample does not provide a precise representation of the population parameter estimate, whereas a narrow confidence interval demonstrates a greater degree of precision. The model validation part of this paper is saying that large/wider confidence interval is obtained for the proposed model and the model is good one. This totally opposite of the general existing process.*

Response 1-3: We agree that a large confidence interval does not guarantee that the model is a better fit. In the revised manuscript, in accordance with **Comments 2-3** from Reviewer #2, we have performed the residual analysis for the proposed and Poisson process models. The residual analysis is a way to assess the goodness of fit of statistical models. As a result, we have newly found that the proposed model fits the data better.

Revised area in the manuscript:
- Lines 280-303
- Figure 7
- Reference [47]

Comment 1-4: *Also, the prediction process is not well defined.*

Response 1-4: To clarify the prediction process, we have visualized the overall structure of the model validation and injury prediction processes in Figure 4. In addition, we have explained the prediction model, input, and output data of the prediction process.

Revised area in the manuscript:
- Lines 231-233, 334-338
- Figure 5

Comment 1-5: *Why validation is done only at 5% and 10% level of significance? Why not for the 1%?*

Response 1-5: We used 5% and 10% levels of significance in accordance with some references. However, 1% and 5% levels are more typical as the reviewer commented. Therefore, we have changed the levels of significance to 1% and 5% in the revised manuscript.

Revised area in the manuscript:
- Line 278
- Figure 6

Minor comment:

Comment 1-6: *The term operations research is mentioned in "Keywords"—what does this concept mean here?*

Response 1-6: Operations research is a discipline that deals with the development and application of analytical methods to improve decision-making. In this sense, our method, which supports making a risk-based injury prevention scenario is one of the research topics of operations research. Therefore, we added this term to the keywords. In the revised manuscript, we have briefly mentioned this point.

Revised area in the manuscript:

- Lines 405-407

Response to Reviewer #2's comments:

Comment 2-0: *The authors present an interesting application of the Hawkes process in modeling the occurrence of kyujo across sumo players' careers.*

Response 2-0:

First of all, thank you very much for reviewing the manuscript and giving us valuable comments. Our answer to your comment is given in the following. Almost all the changes in the manuscript (except for deletions) have been highlighted in yellow.

Major concern:

Comment 2-1: *Since the authors use maximum likelihood estimation, it should be possible to provide basic results for asymptotic normality such as standard errors and Wald-based tests. See, e.g., Serfling (2009) for basic theoretical discussion. Most statistical packages that implement nonlinear estimation should also provide these outputs.*

Response 2-1: We have calculated standard errors of estimates by using the Hessian matrix of the log-likelihood function and have performed the Wald test for them.

Revised area in the manuscript:

- Lines 246-249
- Table 1
- Reference [45]

Comment 2-2: *The kyujo data are subject to censoring. It is unclear what effect this censoring has on the estimation of the underlying failure model. The authors should investigate this to at least some degree. It seems reasonable that with multiple events per player that the effects might be minimal. Is it possible to account for the censoring process in the model estimation?*

The kyujo data appear to be at least partially subject to informative censoring. That is, while players most likely end their careers for a variety of reasons, it seems that the injuries leading to kyujo or repeated kyujo are likely the proximal cause in many cases. In the survival analysis field, this type of informative censoring can sometimes be dealt with via the method of inverse probability of censoring weights (IPCW).

One relatively simple way to handle this would be to set up simulation studies that implement more or less informative censoring. For estimation performance, it would be of interest anyway to see how well the model performs in retrieving model parameters in simulation study.

Response 2-2: We agree that the *kyujo* data are subject to censoring by retirements. It is reasonable

that with multiple events per player, the effects may be minimal. For this reason, we have mentioned this censoring process in the sections on injury data collection and method of maximum likelihood. In addition, we have found that 88% of the wrestlers had one or more *kyujo* occurrences until they retired. This indicates that the effects of censoring may be minimal. Therefore, we assumed that a retirement can be regarded as a *kyujo* occurrence and ignored the censoring. We have discussed that this assumption does not affect the performance of the injury prediction in the section on model validation.

Moreover, we have mentioned that one may use the inverse probability of censoring weights (IPCW) method [32] to handle censoring or copula-based approaches [33] to consider the dependence between *kyujo* occurrences and retirements.

Revised area in the manuscript:

- Lines 108-119, 222-227, 326-330
- Figure 1
- References [32, 33]

Comment 2-3: *The authors write (Line 247) that "the proposed model fits Data-A better than the Poisson process model". This is not really immediately evident from the inspection, given the noise in the data toward the tail end of Figure 4. Is there any way of defining residuals for this model, perhaps similarly to those based on counting processes?*

Response 2-3: We have deleted the corresponding sentence because the result of fitting the confidence intervals was not always evident as the reviewer mentioned. In addition, in accordance with your good suggestion, we have performed residual analysis for the prediction models. As a result, we have newly found that the proposed model fits the data better.

Revised area in the manuscript:

- Lines 280-303
- Figure 7
- Reference [47]

Comment 2-4: *If the authors can devise a calibration plot similar to those found in logistic regression, it might be useful not only in displaying the predictive power but also in evaluating goodness of fit.*

Response 2-4: We have performed calibration for the proposed model. As a result, we have found that the proposed model well predicts the actual *kyujo* occurrences on average.

Revised area in the manuscript:

- Lines 304-325
- Figure 8
- Reference [48]

Comment 2-5: *In the Data-B model fit, the presentation of the predicted number versus the actual number of kyujo is somewhat lost in the text. The text from Lines 276-282 is somewhat opaque. What is being said here? It seems like some of the players with higher predicted risk did in fact become kyujo, but it requires additional work by the reader to figure it out. It is not clear that a confidence interval for number of kyujo is highly informative, but one could construct one from the predicted distribution.*

Response 2-5: Here, our intention is to summarize the injury risks of sumo wrestlers in the grand sumo tournament. Such a summary is helpful for their coaches to understand an overview of wrestlers' injury risks. We have mentioned this point in the revised manuscript. In addition, in accordance with your good suggestion, we have added the confidence interval of the number of wrestlers who are prone to *kyujo* in the tournament.

Revised area in the manuscript:

- Lines 360-367

Comment 2-6: *This general model structure is also handled in survival analysis under the guise of multiple events, although typically without assuming intensity changes as the Hawkes process does. However, one could also implement a time-varying covariate that captured the number of previous kyujo as an approximation. See, e.g., Therneau and Grambsch (2000) for discussion of this model as well as multi-state models which could also be of interest. Could the authors comment briefly on this?*

Response 2-6: Thank you for your suggestion. We have mentioned that multistate models are an alternative way to describe multiple events and are helpful for future work in Conclusion.

Revised area in the manuscript:

- Lines 436-442
- References [49, 50]

Comment 2-7: *Line 200: Change to "subsection".*

Response 2-7: This has been done.

Revised area in the manuscript:

- Line 215
